# Customized binary and multi-level HfO_2−x_-based memristors tuned by oxidation conditions

**DOI:** 10.1038/s41598-017-09413-9

**Published:** 2017-08-30

**Authors:** Weifan He, Huajun Sun, Yaxiong Zhou, Ke Lu, Kanhao Xue, Xiangshui Miao

**Affiliations:** 1 School of Optical and Electronic Information, Huazhong University of Science and Technology, Wuhan, 430074 China; 2 Wuhan National Laboratory for Optoelectronics, Wuhan, 430074 China

## Abstract

The memristor is a promising candidate for the next generation non-volatile memory, especially based on HfO_2−x_, given its compatibility with advanced CMOS technologies. Although various resistive transitions were reported independently, customized binary and multi-level memristors in unified HfO_2−x_ material have not been studied. Here we report Pt/HfO_2−x_/Ti memristors with double memristive modes, forming-free and low operation voltage, which were tuned by oxidation conditions of HfO_2−x_ films. As O/Hf ratios of HfO_2−x_ films increase, the forming voltages, SET voltages, and R_off_/R_on_ windows increase regularly while their resistive transitions undergo from gradually to sharply in ***I***/***V*** sweep. Two memristors with typical resistive transitions were studied to customize binary and multi-level memristive modes, respectively. For binary mode, high-speed switching with 10^3^ pulses (10 ns) and retention test at 85 °C (>10^4^ s) were achieved. For multi-level mode, the 12-levels stable resistance states were confirmed by ongoing multi-window switching (ranging from 10 ns to 1 μs and completing 10 cycles of each pulse). Our customized binary and multi-level HfO_2−x_-based memristors show high-speed switching, multi-level storage and excellent stability, which can be separately applied to logic computing and neuromorphic computing, further suitable for in-memory computing chip when deposition atmosphere may be fine-tuned.

## Introduction

In order to overcome the physical limit in the scaling-down of traditional memories, memristor^1, 2^ is considered to be a promising next generation non-volatile memory^3^ for its high speed^4^, low power consumption^5^, multi-level data storage^6^ and so forth. In various applications, there are different pursuits for the device performances. For example, logic computing^7, 8^ requires high speed and large R_off_/R_on_ window, which could be considered as a demand of binary mode. On the other hand, the potentials of multi-level storage and low power consumption are considered to be crucial for high-density memory^9, 10^ and neuromorphic computing^11–14^, which prefer the multi-level mode instead. Aiming at these two distinct applications, it is natural that different materials may be utilized to meet their particular requirements in the performance. Nevertheless, considering the compatibility to standard CMOS flowline, hafnium oxide^15^, which has already found its way as high-k gate dielectrics in CMOS transistors, is of special technological interest. It is highly worthwhile to examine the possibility of employing hafnium oxide for both applications.

Currently, both binary mode with sharp resistive transition and multi-level mode with gradual resistive transition have been observed in various memristors^16–21^. Nanosecond binary switching and multi-level operation by modulating SET current and RESET voltage have been reported^22–24^. However, such *I*/*V* amplitude modulations of multi-level operation are unsuitable for modern circuit applications and they show no advantage in operation voltage scaling-down if lower power supply is further required^25, 26^. For instance, 200 ns pulse modulation was used to achieve four-level data storage by complex Gd doping into HfO_2−x_ process^27^. But for in-memory computing chip^28^, this is not the best solution taking into account the complexity of process and the limited number of levels. Therefore, the simple unified material process to customize double modes for practical applications is of great significance. On the one hand, the unified material process to achieve different functions in one chip is the general trend with the development of in-memory computing chips. On the other hand, it is difficult for a universal type memristor to manifest extreme performance in a certain aspect, since it ought to achieve a trade-off in all device parameters. In the premise of compatibility with CMOS^29, 30^, customized memristors, however, can perfectly meet practical demands and improve the specificity and efficiency of chips accordingly. Further, as the mobile terminal becomes widespread, low operation voltage in memory devices turns out to be more energy-efficient^31–34^. Thus, the forming-free^35–37^ and low operation voltage were pressing issues for commercialization of memristor. Because of the natural consistency between the low operation voltage and high oxygen vacancies concentration in the sub-stoichiometric memristor^38, 39^, it is worthwhile to further explore the sub-stoichiometric devices to meet the customized demands. S. U. Sharath *et al*.^40^ proposed a scheme towards forming-free resistive switching in oxygen engineered HfO_2−x_. However, sub-stoichiometric films tuned by the oxidation conditions and further research of customized memristor with binary and multi-level modes were not carried out. As described above, it is desirable to customize memristor with double modes, forming-free and low operation voltage in unified HfO_2−x_ material.

In this study, we fabricated an 8 × 8 Pt/HfO_2−x_/Ti crossbar array. By simply adjusting the O_2_/Ar ratio^36, 37^ of reactive sputtering in unified HfO_2−x_ material, we obtain different O/Hf ratios of HfO_2−x_ films. For film composition, the peak area ratio and binding energy of O and Hf from XPS spectrograms are crucial to confirm these O/Hf ratios. For conduction mechanism, Ohmic conduction and Schottky emission are expected to confirm at different states by *I*/*V* and $\mathrm{ln}\,I/\sqrt{V}$ curves fitting. For electrical characteristics, the distinctions in bipolar resistive behaviors of these memristors were researched in *I*/*V* sweep mode. Further, the voltage differences of resistive transitions (from HRS to LRS) were calculated for the next customized mode. For the binary mode, speed and stability are critical properties. In the pulse modulation, the sharp resistive transitions were realized by high-speed response (10 ns, 10^3^ pulses) and device stability was verified by retention test^41^ (85 °C, >10^4^ s), respectively. For the multi-level mode, the potential of multi-level storage can be characterized by compliance current modulation. Further, given practical circuits, the stable 12-levels resistance states were achieved by ongoing multi-window switching of different pulse widths.

## Results

### Characterization of the films and devices

**Figure 1:**
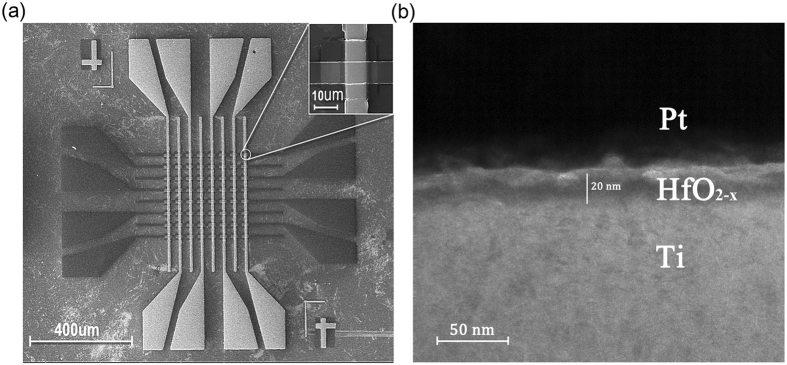
Electrical microscopy of the HfO_2−x_-based memristor. (**a**) SEM image of 8 × 8 Pt/HfO_2−x_/Ti crossbar array, the single device in enlarged part (scale bar:10 μm). (**b**) Cross-sectional TEM image with 20 nm HfO_2−x_ layer after repeated switching cycles.

In order to characterize the microstructure of the Pt/HfO_2−x_/Ti crossbar array, the electrical microscopy was utilized. An 8 × 8 crossbar array in MIM structure is shown in Fig. 1(a) by the scanning electron microscope(SEM). The HfO_2−x_ film deposited by adjusting the O_2_/Ar ratio is the functional layer, whose single device is observed in the enlarged part. Obviously, the vertical cross sections are the electrodes, where the brighter is Pt and the other is Ti. The sharp interfaces between the HfO_2−x_ layer and two electrodes were clearly identified in Fig. 1(b), characterized by transmission electron microscopy (TEM). The interfaces of 20 nm HfO_2−x_ film are apparent after repeated switching cycles^42, 43^.

**Figure 2:**
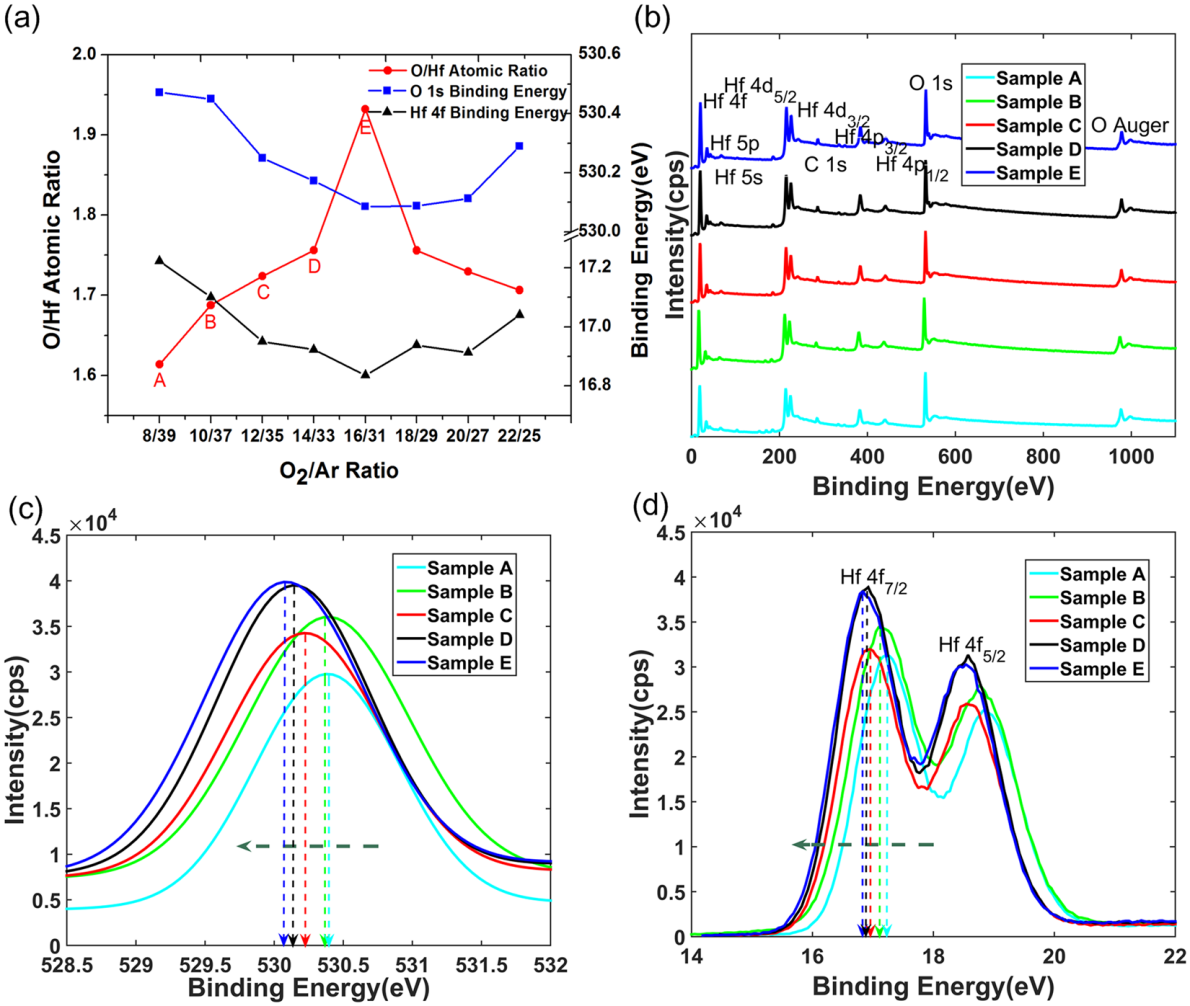
O/Hf ratios and XPS spectrograms of HfO_2−x_ memristors with increasing O_2_/Ar ratios. (**a**) The O/Hf ratio, O 1 s binding energy and Hf 4 f binding energy change regularly with increasing O_2_/Ar ratios. Among them, the O/Hf ratios initially increase and then decrease, while O 1 s and Hf 4 f binding energy show the opposite trend in the corresponding interval. (**b**) The XPS spectrograms of samples A-E are compared and their component of key peaks are labeled. (**c**,**d**) With the increasing O/Hf ratios, The O 1 s and Hf 4 f respectively move to the low binding energy direction.

To quantitatively analyze the atomic ratio O/Hf in the HfO_2−x_ functional layers with respect to the O_2_/Ar ratio of reactive sputtering, the X-ray photoelectron spectroscopy(XPS) measurement was utilized. Figure 2(a) shows the two-coordinate curves for O/Hf ratio, O 1 s binding energy and Hf 4 f binding energy with O_2_/Ar ratios increasing progressively. The left coordinate shows the O/Hf ratios, which increase initially and then decrease after passing through a critical 16/31 O_2_/Ar ratio. In the former part, due to the insufficient oxygen content in the reaction atmosphere, the O content in the resulting HfO_2−x_ film is growing up along with the increasing O_2_ ratios. Yet, with the rapid increasing O_2_ and the decreasing Ar involved in ionization, the probability of oxygen molecules ionization decreases, yielding lower O content in the samples even at high O_2_/Ar ratios in the latter part. The coordinate of the right is the trend of O 1 s and Hf 4 f binding energy, which shows the opposite trend in the corresponding interval compared with O/Hf ratio. Both the Hf 4 f and O 1 s peaks move to the low binding energy direction when the O/Hf ratio approaches to the ideal stoichiometric ratio, as shown in Fig. 2(c,d). This indicates that the degree of oxidation in HfO_2−x_ is improved and the number of oxygen vacancies in the film are reduced, which is consistent with the result of Pereira *et al*.^21, 44^. The memristors corresponding to the first five O/Hf ratios in the former part are defined as samples A-E. The XPS spectrograms of samples A-E are compared and their component of key peaks are labeled in Fig. 2(b). Table 1 summarizes the XPS features of the HfO_2−x_ films.

**Table 1:** O/Hf ratio, O 1 s and Hf 4 f binding energy of HfO_2−x_ films tuned by adjusting the O_2_/Ar ratio of reactive sputtering.

Samples	O_2_/Ar Ratio	O/Hf Ratio	O 1 s Binding Energy	Hf 4 f Binding Energy
A	8/39	1.61	530.472	17.222
B	10/37	1.69	530.450	17.100
C	12/35	1.72	530.250	16.950
D	14/33	1.76	530.173	16.923
E	16/31	1.93	530.086	16.836

### Forming-free, low operation voltage and other bipolar behaviors

**Figure 3:**
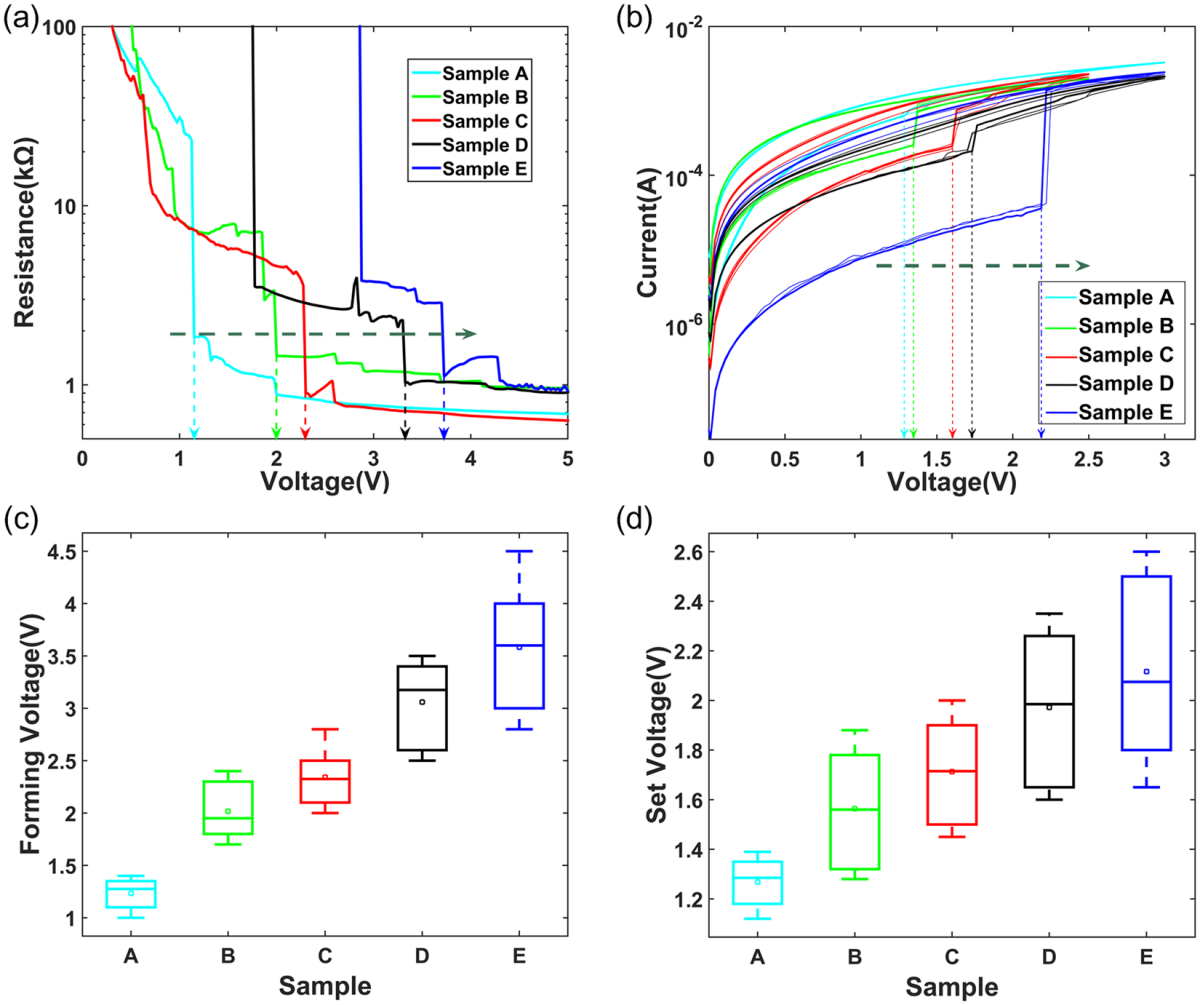
The forming voltages and SET voltages of samples A–E. (**a**) As the O/Hf ratio approaches to stoichiometric ratio, the forming voltages of the samples gradually increase. (**b**) The SET voltages of the samples are increasing similar to the forming voltages. The sample A is a forming-free device as the forming voltage is less than SET voltage. (**c**,**d**) The statistical distribution of forming voltages and SET voltages.

Figure 3(a) shows the forming process of the samples, whose resistances are reduced from the initial meg-ohm level to several thousands of ohms. Obviously, as the O/Hf ratios increase (samples A to E), the forming voltages (1.1 V, 2 V, 2.3 V, 3.3 V and 3.7 V) of the samples increase. It is foreseeable that electroforming requires higher operation voltages when the inherent oxygen vacancies decrease (O/Hf ratios increase). Similar to the forming voltage, SET voltages (1.3 V, 1.4 V, 1.6 V, 1.7 V and 2.2 V) also have the same law shown in Fig. 3(b). Comparing the two voltages, sample A is a forming-free device as the forming voltage is less than SET voltage. For the other samples, the SET voltages are less than the forming voltages and show an inherent increase upon the O/Hf ratio. Figure 3(c,d) show the statistical distribution of forming and SET voltages after repeated responses. From samples A to E, the box patterns show that the fluctuations of forming and SET voltages become larger. Even so, the maximum fluctuations of forming and SET voltages are not more than 2 V and 1 V.

**Figure 4:**
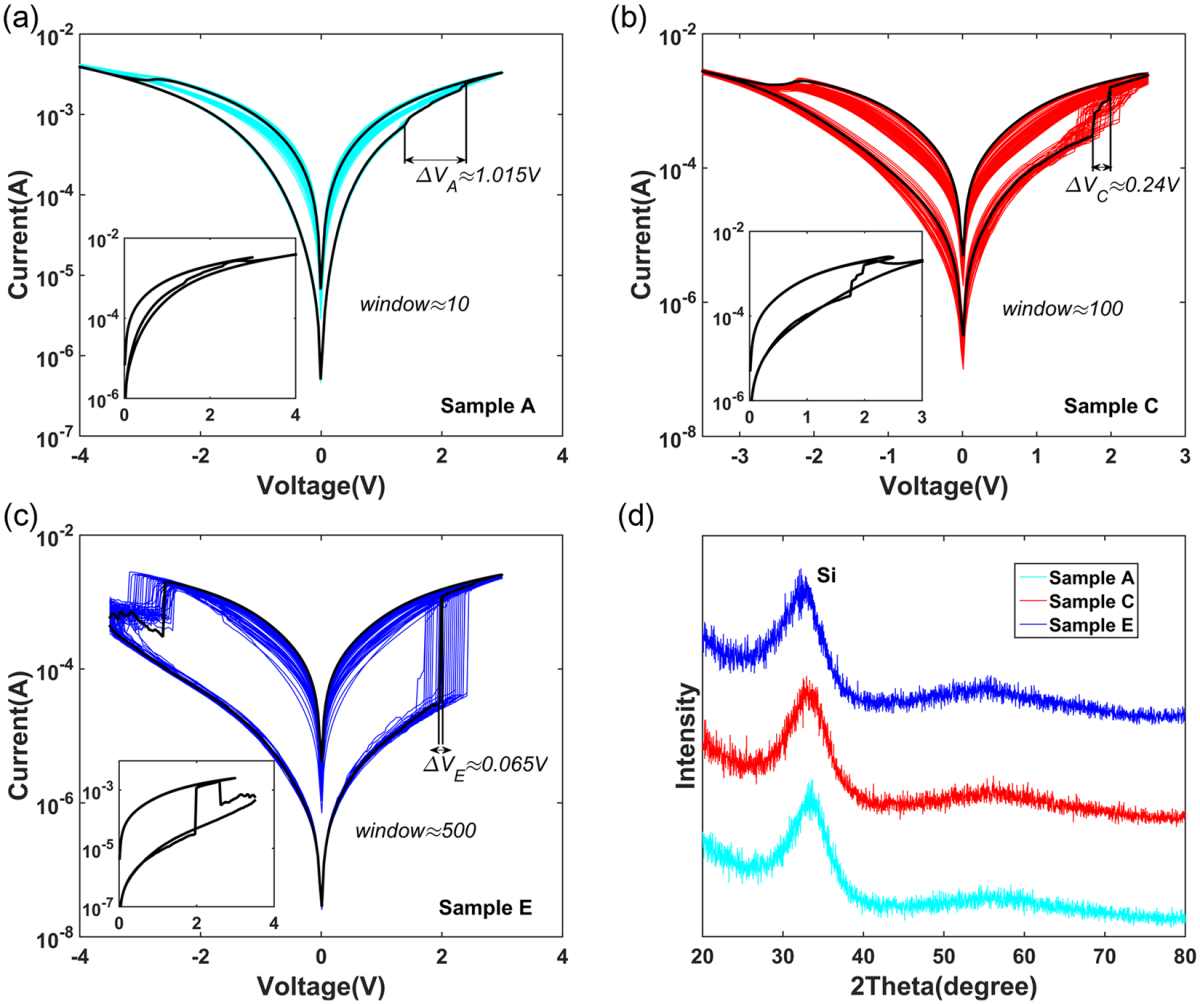
The ***I***/***V*** characteristics of samples A, C, E and X-ray diffraction patterns of HfO_2−x_ films. (**a**,**b**,**c**) The R_off_/R_on_ windows of samples A, C and E are gradually increasing. In addition, the resistive transitions of the samples become much sharper and voltage differences (ΔV_A_, ΔV_C_ and ΔV_E_) are drastically reduced with increasing O/Hf ratios in the black curves. The black curves are shown in the inset with horizontal coordinates of positive voltage and absolute negative voltage. (**d**) The XRD patterns show that all HfO_2−x_ films are amorphous.

Figure 4(a,b,c) shows the I/V hysteresis loops for about 50 cycles in samples A, C and E, whose R_off_/R_on_ windows (10, 100 and 500) are drastically increasing. Although higher O/Hf ratios (less inherent oxygen vacancies) lead to higher high resistance state (HRS) and low resistance state (LRS), the increasing R_off_/R_on_ windows indicate a greater increasing of the HRS. Meanwhile, the processes of forming conductive filaments are much longer for the low O/Hf ratio devices, meaning that they reach the low resistance state (LRS) undergoing longer voltage differences (ΔV). The voltage differences of the samples A (ΔV_A_ = 1.015 V), C (ΔV_C_ = 0.24 V) and E (ΔV_E_ = 0.065 V) are drastically reduced along with the increasing O/Hf ratios in the black curves. This phenomenon can be explained by the argument that the SET process of the oxygen vacancies-enriched memristor is a process of thickening the conductive filaments. The low O/Hf ratio memristors start to form the conductive filament at lower voltage and undergo longer voltage differences to reach the LRS. For the high O/Hf ratios, undergoing shorter voltage differences means much sharper for resistive transitions. They start to flip over at higher voltages (higher energy) and form the conductive filaments from none to multiple roots instantly. In this paper, customized binary and multi-level modes are just pointed out based on the difference between rapid voltage jump and controllable thickening process. As a comparison, these black curves with typical resistive transitions are shown in the inset of Fig. 4(a,b,c) with horizontal coordinates of positive voltage and absolute negative voltage. The asymmetry of the devices can be found by comparing their positive and negative voltage jump points. The XRD patterns indicate that all HfO_2−x_ films of samples are amorphous shown in Fig. 4(d). As S. U. Sharath *et al*.^45^ reported the representative phase structure of hafnium oxide films, the growth temperature and oxidation conditions have a crucial effect on the structure of HfO_2−x_ films. The growth at room temperature dominates the amorphous structure, while the oxygen vacancies further aggravate it.

### The curves fitting and mechanism analysis

**Figure 5:**
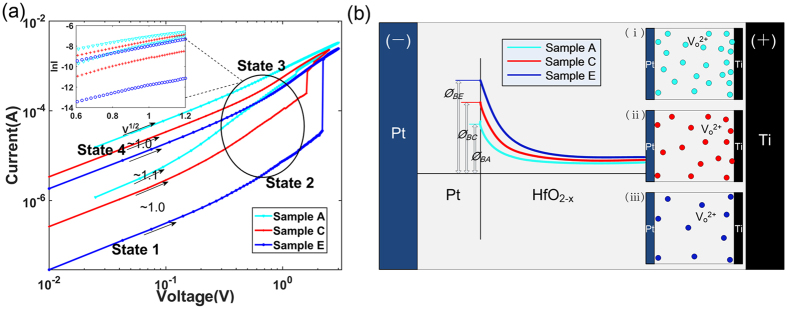
The curve fitting and mechanism analysis of the samples A, C and E. (**a**) The slopes of samples A, C and E at low voltage region are approximately 1 in the double logarithmic coordinates of SET process and further indicate the Ohmic conduction at state 1 and state 4. The inset shows linear fits of the $\mathrm{ln}\,I/\sqrt{V}$ at high voltage region. State 2 is the Schottky emission and state 3 is actually the result of combination of Ohmic conduction and Schottky emission. (**b**) The Schottky barrier of the initial state is shown schematically. The Schottky barriers of the Pt/HfO_2−x_ interface gradually increase from samples A to E. The inset (i–iii) show the reduction of inherent oxygen vacancies in the non-intrinsic HfO_2−x_ films. And the amount of oxygen vacancies at the Ti/HfO_2−x_ interface is much larger than those at the other position of the films.

Comparative SET processes are carried out to study the conduction mechanism. As shown in Fig. 5(a), four *I*/*V* states (HRS in low voltage, HRS in high voltage, LRS in high voltage and LRS in low voltage) are named state 1, state 2, state 3 and state 4 for simple notation. The slopes of samples A, C and E at state 1 are approximately 1 in the double logarithmic coordinates, which indicate that the memristors are in Ohmic conduction at this state shown in Fig. 5(a). As the voltage increases, all curves bend upward at state 2. Considering that Pt has high work function (5.65 eV), which is much higher than the electron affinity of HfO_2−x_, the Schottky barrier is inevitably formed at the Pt/HfO_2−x_ interface. The inset shows linear fits of $\mathrm{ln}\,I/\sqrt{V}$ to confirm the Schottky emission at state 2^30, 46–48^. The previous *I*/*V* hysteresis loops show obvious asymmetry in the inset of Fig. 4(a,b,c), further supporting the interface-limited Schottky emission mechanism. This phenomenon can be explained by the insufficient energy for the electron to surmount the barrier (emission threshold) at state 1. When the SET voltage exceeds the emission threshold, the Schottky emission exponentially increases and dominates the conduction mechanism. However, when the device transforms from the HRS to the LRS, conductive filaments are formed and Ohmic conduction are established. As most research works reported the presence of multiple filaments in oxide memristors^49–51^, we envisage a scenario as below. Only a few conductive filaments have punched through the electrodes, while most filaments remain in the partial form, such that the state 3 is actually the result of the combination of Ohmic conduction and Schottky emission. As the SET voltage is less than the threshold, the Schottky emission is gradually diminished and the Ohmic conduction dominates the conductive mechanism again at state 4.

Figure 5(b) shows the initial Schottky barriers of the samples A, C and E. Although Pt electrodes of the samples A, C and E have same work function, the electron affinities of non-intrinsic HfO_2−x_ films are different due to their dispersed oxygen vacancies concentrations. Therefore, the Schottky barriers of the Pt/HfO_2−x_ interfaces gradually increase from samples A to E, which is also responsible for the increasing forming and SET voltages. The inset (i)(ii)(iii) show the reduction of inherent oxygen vacancies in the non-intrinsic HfO_2−x_ films of samples A, C, and E. The distinctions of inherent oxygen vacancies are tuned by oxidation conditions. It should be noted that the amount of oxygen vacancies at the Ti/HfO_2−x_ interface is much larger than those at the other position of the films.

The microscopic mechanism of the Pt/HfO_2−x_/Ti memristor was summarized as follows. On the one hand, due to the active characteristic of Ti, oxygen atoms will be extracted from the HfO_2−x_ films to form Titanium oxide^52^. On the other hand, the non-intrinsic HfO_2−x_ films have several oxygen vacancies in a certain extent. The combination of the two reasons makes the devices contain oxygen vacancies in initial state, providing a congenital condition to reduce forming and SET voltages. When the positive voltage is applied to the Ti electrode, the Ti/HfO_2−x_ interface oxidation is enhanced, at the same time the oxygen vacancies in the HfO_2−x_ film migrate toward the Pt electrode. A large amount of oxygen vacancies accumulate at the Pt/HfO_2−x_ interface to lower the valency of Hf^25^ and form oxygen vacancies conductive filaments through the Schottky barrier of Pt/HfO_2−x_ interface, transforming the devices into the LRS. When the negative voltage is applied to the Ti electrode, the oxygen ions migrate toward the Pt electrode, i.e., the oxygen vacancies are considered to be extracted from the Pt/HfO_2−x_ interface. At the interface, the transition proceeds in the following sequence: (i) the concentration of oxygen vacancies decreasing; (ii) the valency of Hf increasing; (iii) the conductive filaments breaking, eventually transform the devices back to the HRS.

### Binary and multi-level memristive modes

**Figure 6:**
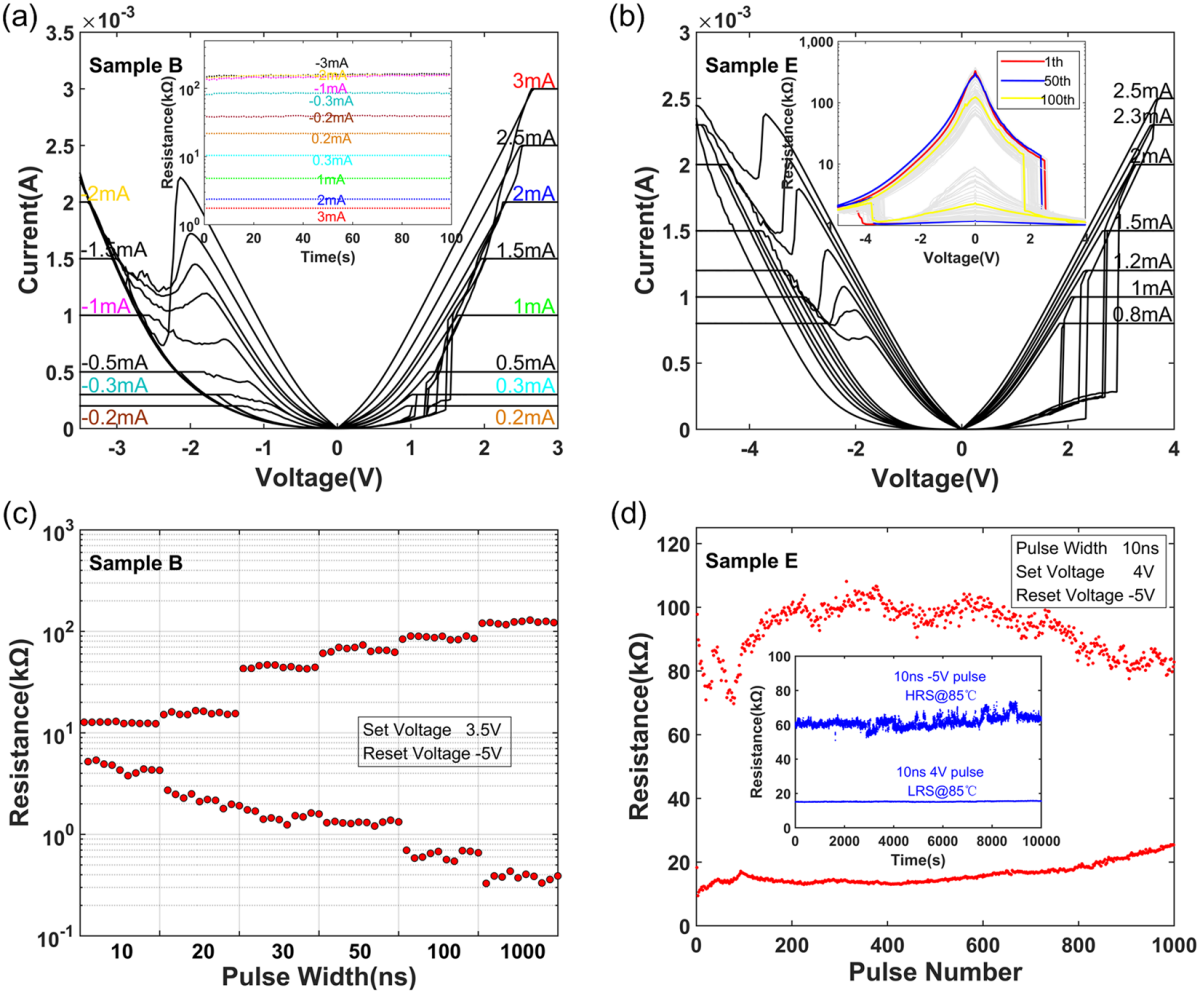
Binary and multi-level modes corresponding to samples B and E. (**a**) Sample B can be located at different resistance states by setting compliance currents. The inset shows stable R/T curves after above compliance current operations. (**b**) Sample E exhibits dense ***I***/***V*** curves near the 0 V voltage. The inset shows the device transforms obviously at 2 V and −3.5 V of 100 cycles ***I***/***V*** hysteresis loops. (**c**) Multi-level resistance states (12 levels) of Sample B were realized by ongoing multi-window switching of different pulse widths, and each pulse completed 10 switching cycles. (**d**) Sample E realized high-speed (10 ns) switching response in 10^3^ pulses. The inset shows retention characteristics at 85 °C (>10^4^ s) and the blue curves were tested after the 999th and 1000th high-speed pulses.

The distinctions in resistive transition of different O/Hf ratio memristors are expected to customize operation modes. The process of resistive transition is gradual for low O/Hf ratios, while the high O/Hf ratios transform sharply, which has been shown above. Taking into account the demand of R_off_/R_on_ windows, Samples B and E are selected as representatives of low and high O/Hf ratios to study. The compliance currents are applied to observe whether they have stable multi-level resistance states. Sample B can be stably located at different resistance levels by setting different compliance currents as shown in Fig. 6(a). The discrete resistive transition of SET and RESET processes show that the memristor has the multi-level potentials. The inset further shows stable resistance states (from 1 kΩ to 200 kΩ), which are obtained in above different compliance currents. In addition, this memristor has the advantages of low forming voltage (2 V) and SET voltage(1.4 V), which can effectively reduce power consumption.

In Fig. 6(b), Sample E shows dense *I*/*V* curves near the 0 V voltage even if different compliance currents are set. The sharp resistive transitions would contribute to the high-speed switch response^53, 54^. The inset shows the device transforms obviously in SET (at 2 V) and RESET (at −3.5 V) processes of 100 cycles *I*/*V* hysteresis loops, which further proves that the memristor prefers binary mode. Compared to multi-level mode, the binary mode sacrifices higher operation voltage, but larger window and faster response can be obtained instead.

The high-speed pulse test is further completed in order to make it close to the practical customized modes and demonstrate their speed advantage. Figure 6(c) shows different resistance states (12 levels) by ongoing multi-window switching of different pulse widths. The pulses of 10 ns, 20 ns, 30 ns, 50 ns, 100 ns and 1 μs are applied to Sample B, and each pulse has completed 10 switching cycles. The switching response in each pulse width exhibits excellent stability, which can be characterized by stable resistance states. The R_off_/R_on_ windows extend upward (HRS) and downward (LRS) with increasing pulse widths, which is feasible to realize multi-level data storage.

As a contrast, Fig. 6(d) shows the excellent binary performance of sample E. It can achieve approximate 10 times R_off_/R_on_ window and 10^3^ serial tests^55, 56^ in high-speed (10 ns) pulses. Compared with the 10 ns-response of sample B, the larger window and longer test times of sample E further support its superiority for binary mode. The inset shows the retention tests at 85 °C (>10^4^ s). The LRS and HRS retention characteristics after the 999th and 1000th high-speed pulses are shown in blue curves. The LRS exhibits excellent stability and the HRS fluctuates slightly after the practical high-speed pulse operation.

## Discussions and Conclusions

This work is guided by the conduction mechanisms due to oxygen vacancies. Because of the natural consistency between high oxygen vacancies concentration and low operation voltage in sub-stoichiometric memristor, it is worthwhile to further explore the sub-stoichiometric devices to realize low voltage operation. Further, due to the distinction in inherent oxygen vacancies concentration, these sub-stoichiometric memristors show differences in the process of forming the conductive filaments. It is conceivable that these differences may be utilized to achieve binary and multi-level modes, which will be beneficial to pursuing different performances in memristor. For these different fields, the characteristic tests of relevant applications are thus completed.

In summary, customized HfO_2−x_ memristor is constructed to realize binary and multi-level modes tuned by oxidation conditions. The O/Hf ratios of the HfO_2−x_ films are modulated by adjusting the O_2_/Ar ratio of reactive sputtering. Forming-free and low operation voltage were confirmed for low O/Hf ratio memristors at the same time large R_off_/R_on_ window and sharp resistive transition were found for high O/Hf ratio memristors. Further, the sharp and gradual resistive transitions can be utilized to customize binary and multi-level modes, respectively. For binary mode, 10^3^ pulses (10 ns) are serially applied to realize resistive switching of approximate 10 times R_off_/R_on_ window, which still maintains excellent stability at 85 °C (>10^4^ s). For multi-level mode, 12-levels resistance states and 10 switching cycles of each pulse width have been completed by ongoing multi-window switching of incremental pulse widths. Besides, Ohmic conduction and Schottky emission mechanisms were confirmed at different states. Taking into account the distinctions of applied mode and operation voltage, these binary and multi-level memristors in unified HfO_2−x_ material can be applied to logic computing, neuromorphic computing and in-memory computing chip.

## Methods

### Device fabrication

A silicon substrate with a thin silicon dioxide layer was pre-cleaned to avoid contamination from the ambience. The bottom electrode pattern was lithography by a well-designed mask, and 120 nm Ti thin film was deposited by DC sputtering. The HfO_2−x_ functional layer of the small square pattern was likewise lithography after the previous lift-off process. 20 nm HfO_2−x_ films were deposited by adjusting the O_2_/Ar ratio of reactive sputtering to achieve customized memristor. Finally, after another lift-off and lithography process, a 120 nm Pt film as top electrode was deposited by DC sputtering and then lifted off last time. The finished memristor of 8 × 8 Pt/HfO_2−x_/Ti crossbar array was completed by binding and used for various tests.

### Device structure and film composition

The microstructure of the device was monitored by scanning electron microscope (SEM) and the cross-sectional structure was monitored by transmission electron microscopy (TEM) using FEI Quanta 200 and Tecnai G2 20 equipment, respectively. The amorphous structure of the HfO_2−x_ films was examined by X-ray diffraction (XRD) using PANalytical PW3040-60 MRD. The atomic concentration in the HfO_2−x_ films was characterized by X-ray photoelectron spectroscopy (XPS) using an AXIS-ULTRA DLD-600W system.

### Electrical characterization

The DC electrical characteristics were carried out with an Agilent B1500A semiconductor parameter analyzer in *I*/*V* sweep mode. And the high-speed pulse response was collected by Keithley 4200 semiconductor parameter analyzer. The retention tests at 85 °C were performed on a Cascade Microtech M150 probe system equipped with a thermal chuck (0.1 °C accuracy), which was periodically (every one second) monitored by Agilent B1500A with a low read voltage (0.1 V) in *I*/*V*-*t* Sampling mode. For all the electrical measurements, the top electrodes (Pt) were grounded while the bottom electrodes (Ti) were biased.

## References

[1] Chua L (1971). Memristor-the missing circuit element. IEEE Transactions on Circuit Theory.

[2] Strukov DB, Snider GS, Stewart DR, Williams RS (2008). The missing memristor found. Nature.

[3] Chen Y (2003). Nanoscale molecular-switch crossbar circuits. Nanotechnology.

[4] Torrezan AC, Strachan JP, Medeiros-Ribeiro G, Williams RS (2011). Sub-nanosecond switching of a tantalum oxide memristor. Nanotechnology.

[5] Pickett MD, Williams RS (2012). Sub-100 fJ and sub-nanosecond thermally driven threshold switching in niobium oxide crosspoint nanodevices. Nanotechnology.

[6] Pan F, Gao S, Chen C, Song C, Zeng F (2014). Recent progress in resistive random access memories: materials, switching mechanisms, and performance. Mater. Sci. and Eng. R.

[7] Zhou Y (2015). 16 Boolean logics in three steps with two anti-serially connected memristors. Appl. Phys. Lett..

[8] Zhou, Y.-X. *et al*. Nonvolatile reconfigurable sequential logic in a HfO_2_ resistive random access memory array. *Nanoscale* (2017). 10.1039/c7nr00934h 28261713

[9] Yang YC, Pan F, Liu Q, Liu M, Zeng F (2009). Fully room-temperature-fabricated nonvolatile resistive memory for ultrafast and high-density memory application. Nano Lett..

[10] Wang Y (2009). Investigation of resistive switching in Cu-doped HfO_2_ thin film for multilevel non-volatile memory applications. Nanotechnology.

[11] Li Y (2014). Activity-dependent synaptic plasticity of a chalcogenide electronic synapse for neuromorphic systems. Sci. Rep..

[12] Jiang L (2016). Conductance quantization in an AgInSbTe-based memristor at nanosecond scale. Appl. Phys. Lett..

[13] Jo SH (2010). Nanoscale memristor device as synapse in neuromorphic systems. Nano Lett..

[14] Yang X (2016). Nonassociative learning implementation by a single memristor-based multi-terminal synaptic device. Nanoscale.

[15] Wedig A (2016). Nanoscale cation motion in TaO_x_, HfO_x_ and TiO_x_ memristive systems. Nat. Nanotechnol..

[16] Waser R, Aono M (2007). Nanoionics-based resistive switching memories. Nat. Mater..

[17] Yoshida C, Tsunoda K, Noshiro H, Sugiyama Y (2007). High speed resistive switching in Pt/TiO_2_/TiN film for nonvolatile memory application. Appl. Phys. Lett..

[18] Fang Z (2011). HfO_x_/TiO_x_/HfO_x_/TiO_x_ Multilayer-Based Forming-Free RRAM Devices With Excellent Uniformity. IEEE Electron Device Lett..

[19] Yu S (2010). Improved uniformity of resistive switching behaviors in HfO_2_ thin films with embedded Al layers. Electrochem. Solid-State Lett..

[20] Wang Z (2016). Modulation of nonlinear resistive switching behavior of a TaO_x_-based resistive device through interface engineering. Nanotechnology.

[21] Pereira L, Barquinha P, Fortunato E, Martins R (2005). Influence of the oxygen/argon ratio on the properties of sputtered hafnium oxide. Mater. Sci. and Eng. B.

[22] Lee H (2010). Low-power and nanosecond switching in robust hafnium oxide resistive memory with a thin Ti cap. IEEE Electron Device Lett..

[23] Sheu, S.-S. *et al*. In VLSI Circuits, Symposium on. 82–83 (IEEE) (2009).

[24] Zhao, X. *et al*. Confining cation injection to enhance CBRAM performance by nanopore graphene layer. *Small* (2017). 10.1002/smll.201603948 28234422

[25] Lin Y (2013). Resistive switching mechanisms relating to oxygen vacancies migration in both interfaces in Ti/HfO_x_/Pt memory devices. J. Appl. Phys..

[26] Chen, Y.-S. *et al*. In VLSI Technology, Systems, and Applications, VLSI-TSA'09. International Symposium on. 37–38 (IEEE) (2009).

[27] Gao B (2013). A novel defect-engineering-based implementation for high-performance multilevel data storage in resistive switching memory. IEEE Trans. Electron Devices.

[28] Hamdioui, S. *et al*. In *Proceedings of the Design, Automation & Test in Europe Conference & Exhibition*. 1718–1725 (EDA Consortium) (2015).

[29] Waser R, Dittmann R, Staikov G, Szot K (2009). Redox‐based resistive switching memories–nanoionic mechanisms, prospects, and challenges. Adv. Mater..

[30] Wong H-SP (2012). Metal–oxide RRAM. Proc. IEEE.

[31] Bessonov AA (2015). Layered memristive and memcapacitive switches for printable electronics. Nat. Mater..

[32] Kim Y-M, Lee J-S (2008). Reproducible resistance switching characteristics of hafnium oxide-based nonvolatile memory devices. J. Appl. Phys..

[33] Lee H-Y (2007). Low-power switching of nonvolatile resistive memory using hafnium oxide. Jpn. J. Appl. Phys..

[34] Govoreanu, B. *et al*. In Electron Devices Meeting (IEDM), IEEE International. 31.36. 31–31.36. 34 (IEEE) (2011).

[35] Yang JJ (2009). The mechanism of electroforming of metal oxide memristive switches. Nanotechnology.

[36] Wang S-Y, Lee D-Y, Huang T-Y, Wu J-W, Tseng T-Y (2010). Controllable oxygen vacancies to enhance resistive switching performance in a ZrO_2_-based RRAM with embedded Mo layer. Nanotechnology.

[37] Klein J, Clauson S, Cogan S (1995). Reactive IrO_2_ sputtering in reducing/oxidizing atmospheres. J. Mater. Res..

[38] Yang JJ (2008). Memristive switching mechanism for metal/oxide/metal nanodevices. Nat. Nanotechnol..

[39] Padovani A, Larcher L, Bersuker G, Pavan P (2013). Charge transport and degradation in HfO_2_ and HfO_x_ dielectrics. IEEE Electron Device Lett..

[40] Sharath S (2014). Towards forming-free resistive switching in oxygen engineered HfO_2−x_. Appl. Phys. Lett..

[41] Yang, P.-K. *et al*. A Fully Transparent Resistive Memory for Harsh Environments. *Sci. Rep*. **5** (2015). 10.1038/srep15087 PMC4601025 26455819

[42] Liu S (2016). Eliminating Negative‐SET Behavior by Suppressing Nanofilament Overgrowth in Cation‐Based Memory. Adv. Mater..

[43] Sun H (2014). Direct observation of conversion between threshold switching and memory switching induced by conductive filament morphology. Adv. Funct. Mater..

[44] Dupin J-C, Gonbeau D, Vinatier P, Levasseur A (2000). Systematic XPS studies of metal oxides, hydroxides and peroxides. Phys. Chem. Chem. Phys..

[45] Sharath S (2014). Thickness independent reduced forming voltage in oxygen engineered HfO_2_ based resistive switching memories. Appl. Phys. Lett..

[46] Lu C (2016). Self-compliance Pt/HfO_2_/Ti/Si one-diode–one-resistor resistive random access memory device and its low temperature characteristics. Appl. Phys. Express.

[47] Walczyk C (2011). Impact of Temperature on the Resistive Switching Behavior of Embedded HfO_2_-Based RRAM Devices. IEEE Trans. Electron Devices.

[48] Tran, X. *et al*. In Electron Devices Meeting (IEDM), IEEE International. 31.32. 31-31.32. 34 (IEEE) (2011).

[49] Celano U (2013). Filament observation in metal-oxide resistive switching devices. Appl. Phys. Lett..

[50] Chen J-Y (2013). Dynamic evolution of conducting nanofilament in resistive switching memories. Nano Lett..

[51] Xue K-H (2014). A Combined Ab Initio and Experimental Study on the Nature of Conductive Filaments in Pt/HfO_2_/Pt Resistive Random Access Memory. IEEE Trans. Electron Devices.

[52] Sowinska M (2012). Hard x-ray photoelectron spectroscopy study of the electroforming in Ti/HfO_2_-based resistive switching structures. Appl. Phys. Lett..

[53] Burr GW (2008). Overview of candidate device technologies for storage-class memory. IBM. J. Res. Dev..

[54] Breuer, T. *et al*. Realization of Minimum and Maximum Gate Function in Ta_2_O_5_-based Memristive Devices. *Sci. Rep*. **6** (2016). 10.1038/srep23967 PMC4820708 27046279

[55] Niu, G. *et al*. Geometric conductive filament confinement by nanotips for resistive switching of HfO_2_-RRAM devices with high performance. *Sci. Rep*. **6** (2016). 10.1038/srep25757 PMC4867633 27181525

[56] Jiang, H. *et al*. Sub-10 nm Ta Channel Responsible for Superior Performance of a HfO_2_ Memristor. *Sci. Rep*. **6** (2016). 10.1038/srep28525 PMC4917839 27334443

